# Response of young rice panicles to salt stress: insights based on phenotype and transcriptome analysis

**DOI:** 10.3389/fpls.2024.1451469

**Published:** 2024-10-21

**Authors:** Fanrui Duan, Fugui Wu, Zhen Li, Kai Zhang, Qilin Ma

**Affiliations:** School of Tropical Agriculture and Forestry, Hainan University, Haikou, Hainan, China

**Keywords:** rice, panicle differentiation, salt stress, RNA-Seq, WGCNA

## Abstract

Saline-alkali soils limit rice growth and production. With an increasing global population, enhancing rice salt tolerance is crucial for improving yields in these areas. This study investigated the developmental characteristics of young panicles and pollen fertility in two rice varieties, 58M and 58L, under salt stress. Results showed that 58M had more substantial salt tolerance during panicle development. RNA sequencing of 18 samples from both varieties under high salt stress (0 h, 6 h, and 24 h) identified 469 common differentially expressed genes (DEGs) and 2,308 DEGs between the varieties. Kyoto Encyclopedia of Genes and Genomes (KEGG) pathway enrichment highlighted significant pathways such as phenylpropanoid biosynthesis, protein processing, and flavonoid biosynthesis. Six gene co-expression modules related to salt tolerance were identified, with six candidate genes (*LOC_Os05g38530*, *LOC_Os04g07920*, *LOC_Os12g02105*, *LOC_Os01g06580*, *LOC_Os06g49250*, and *LOC_Os06g48300*) potentially linked to salt tolerance. These findings provide insights into rice salt tolerance mechanisms and offer new genetic resources for breeding salt-tolerant rice.

## 1 Introduction

At present, the world population is approximately 7.95 billion (https://data.worldbank.org/indicator/SP.POP.TOTL), and the annual growth rate is at 0.8% (https://data.worldbank.org/indicator/SP.POP.GROW). Meanwhile, the per capita arable land has decreased by approximately 50% in the past 50 years (https://data.worldbank.org/indicator/AG.LND.ARBL.HA.PC). An increase in population inevitably demands an increase in food production. However, the push for urbanization and industrialization in countries worldwide will inevitably lead to a reduction in arable land. Rice, one of the major staple food crops globally, can only meet the increasing food demand caused by population growth and the land environmental changes due to urbanization by expanding its cultivation to marginal areas such as saline-alkali soil. Approximately 52% of the world’s population lives in the 13 countries most severely affected by soil salinity (Liu et al., 2020). Therefore, research into salt-tolerant rice is an urgent and objective necessity.

Rice’s stress response to salt stress manifests in ion homeostasis, osmotic adjustment, and reactive oxygen species (ROS) scavenging (Ganapati et al., 2022). Ion homeostasis refers to the equilibrium of Na^+^ and K^+^ in high-salt soils, primarily due to the shared transmembrane transport proteins for Na^+^ and K^+^ entering the cell (Greenway and Munns, 1980). Plant osmotic regulation, protein synthesis, cell morphological structure stability, and photosynthesis all require the involvement of K^+^ (Ashraf and Harris, 2004). Excessive accumulation of Na^+^ can cause ion toxicity, leading to decreased K^+^ content. This reduction affects the activity of K^+^-activated enzymes, resulting in the slow growth of rice seedlings (Yang and Guo, 2018). SOS1 is a Na^+^ efflux transporter that, under salt stress, works to expel excessive Na^+^ from the cell, maintaining Na^+^/K^+^ homeostasis (El Mahi et al., 2019). Osmotic adjustment refers to the process where rice roots, facing a significant water potential difference in high-salt environments, promote the biosynthesis and accumulation of compatible osmolytes to balance the internal and external osmotic pressure, preventing cell dehydration and death (Chen et al., 2021). For instance, in rice seedlings under salt stress, the expression of proline biosynthetic genes *OsP5CS1*, *OsP5CS2*, and *OsP5CR* is upregulated, while the proline degradation metabolism genes *OsPDH1* and *OsP5CDH* are downregulated. This indicates that proline accumulation benefits plant osmotic balance (Lv et al., 2014). ROS scavenging refers to the significant accumulation of ROS, such as superoxide anions and hydrogen peroxide, in the rice roots due to salt stress. This accumulation leads to the continuous upregulation of the cell death-related genes *OsKOD1*, *OsHsr203j*, *OsCP1*, and *OsNAC4* (Zhang et al., 2017). The accumulation of ROS activates the plant’s antioxidant system, resulting in the upregulation of antioxidant enzyme-related genes such as superoxide dismutase (SOD), ascorbate peroxidase (APX), and catalase (CAT), thereby decomposing excessive ROS and reducing cell damage (Hasanuzzaman et al., 2021).

In recent years, the high-throughput capabilities of RNA-Seq, which have become increasingly sophisticated, have been used to identify transcription factors and analyze co-expression networks. This has provided a reliable basis for revealing salt tolerance traits in rice and for screening potential candidate genes (Wang et al., 2023). A co-expression network utilizes the characteristics of functionally related genes in biological cells that express in a coordinated manner under specific conditions. This is achieved by calculating the correlation coefficient matrix between the genes and then representing it as a network. This approach allows for the analysis of multiple expression modules and identifying core genes within these modules (Langfelder and Horvath, 2008). By constructing a gene co-expression network under rice salt stress, it is possible to unveil the molecular mechanisms of rice in response to salt stress. This provides potential candidate genes for the breeding of salt-tolerant varieties.

Due to it being a significant food crop, research on the salt tolerance of rice is crucial for increasing rice production area, increasing rice yield, and meeting the food demand of a growing population. There are significant differences in salt tolerance among different rice varieties (Sriskantharajah et al., 2020), and the mechanism of salt tolerance is still unclear. The differentiation and development of young panicles is a critical period in the formation of rice, and it is also the most sensitive period for rice to abiotic stress. Under abiotic stress conditions, rice generally exhibits spikelet degeneration, spikelet abortion (pollen abortion), and poor grain filling (Panda et al., 2016). Salt stress usually reduces the number of spikelets per panicle, leading to spikelet degeneration and pollen sterility, affecting seed setting rate and yield (Razzaq et al., 2020). To achieve a high yield, increasing the number of spikelets per panicle, reducing the number of abortive spikelets, and improving the seed setting rate are key factors during the development of young panicles. Little information is known about salt tolerance-related genes during the differentiation stage of young rice panicles.

Therefore, in Haikou City, Hainan Province, we selected two rice varieties with different levels of salt tolerance for our study. We conducted pollen collection and observation counts under three different concentrations of salt stress and RNA-Seq sequencing at different durations of stress under the same high salt concentration. Through differential expression analysis of transcriptomic sequencing data, clustering analysis of differentially expressed genes (DEGs), gene ontology (GO) and Kyoto Encyclopedia of Genes and Genomes (KEGG) enrichment analysis, and using weighted gene co-expression network analysis (WGCNA) to construct a co-expression network and quantitative reverse transcription PCR (qRT-PCR), we identified several candidate genes for rice salt tolerance. This study provides a theoretical foundation for further understanding the molecular mechanisms of rice salt tolerance and offers genetic resources for improving research on rice salt tolerance.

## 2 Materials and methods

### 2.1 Plant materials and growth conditions

This study selected two rice varieties, 58M with strong salt tolerance and 58L with weak salt tolerance, independently bred in the laboratory at the early stage. The breeding procedures for 58M and 58L are as follows: 58L is the rice variety Haixiang 030 obtained by introducing exogenous reed DNA into indica rice variety 9311 through the pollen tube pathway method. The acquisition of 58M is even more complex. First, Haixiang 030 underwent ethyl methanesulphonate (EMS) mutagenesis breeding to obtain rice varieties 14-90. Then, F1 was obtained by crossbreeding 9311 as the female parent and Brazilian upland rice as the male parent and was used as the female parent. F1 was obtained by crossbreeding 14-90 as the female parent and Yanhui 559 as the male parent and was used as the male parent. After multiple generations of self-pollination, a variety with excellent salt tolerance, namely 58M, was finally selected. Both rice materials were planted at the Agricultural Science Base of the School of Tropical Agriculture and Forestry, Hainan University, Haikou City, Hainan Province, China (110° 19’ E, 20° 03’ N). Well-growing seeds were selected for germination in the experiment. After the four-leaf seedling stage, they were transplanted into partitioned membrane pools with the same number of plants in each partition. Conventional cultivation methods were used for management. We used anhydrous sodium chloride for salt treatment before heading differentiation, with sodium chloride mass fractions of 0%, 0.2%, 0.4%, and 0.6%, which continued until the pollen mother cell formation stage. During the experiment, the salt concentration in the pool was monitored three times a day (morning, noon, and evening) and adjusted promptly to ensure a stable salt concentration. At the same time, some plants were selected for pot experiments for further observation. During the young panicle differentiation stage, the young panicles were peeled off to determine the developmental stage. When the length of the young ear reached 2 cm, a 0.6% NaCl solution was used to treat the young ear with salt stress. Young ear samples were collected at 0h, 6h, and 24h, and quickly frozen in liquid nitrogen for subsequent experiments.

### 2.2 Pollen fertility observation

From each variety under different treatments, three spikelets were randomly selected from the middle part of the panicle. The anthers were peeled off, and the pollen was extracted using tweezers and then smeared onto a glass slide containing a drop of a solution of potassium iodine with iodide. The slide was observed under a microscope (Fahad et al., 2015). Five different fields of view were randomly selected for each sample, and photo records were saved. At the same time, after the rice heading, young ears of each variety treated with different treatments were randomly selected for morphological observation.

### 2.3 RNA extraction, cDNA library preparation, and sequencing

Total RNA was extracted using the OminiPlant RNA Kit (CW2598S) provided by Jiangsu Cowin Biotech Co., Ltd (Taizhou, China). The extracted total RNA was stored at -80°C and transported to Guangzhou Kidio Biotechnology Co., Ltd. (Guangzhou, China) on dry ice for sequencing (Illumina HiSeq X Ten). Eukaryotic mRNA with polyA tails was enriched using magnetic beads with Oligo (dT) and then fragmented by ultrasonication. Using the fragmented mRNA as a template and random oligonucleotides as primers, the first strand of cDNA was synthesized in an M-MuLV reverse transcriptase system. The RNA strand was then degraded with RNase H, and the second cDNA strand was synthesized using DNA Polymerase I and dNTPs. The purified double-stranded cDNA underwent end repair, A-tailing, and adapter ligation. cDNA fragments approximately 200 bp in size were selected using AMPure XP beads, followed by PCR amplification and further purification of the PCR products with AMPure XP beads, resulting in the final cDNA library. The raw data obtained were quality-controlled using FastQC and filtered with trim_galore. The clean data were used for subsequent analyses. Reference genome-based alignment was performed using HISAT2 (Liu et al., 2022) (Reference genome source: http://rice.uga.edu/), sam files were converted to bam files using samtools (Li et al., 2009), and gene expression data matrices were calculated using StringTie for further data analysis (Pertea et al., 2016).

### 2.4 Identification of differentially expressed genes

Since the experimental materials were subjected to a paired-end sequencing strategy, fragments per kilobase of transcript per million mapped reads (FPKM) were chosen over reads per kilobase of transcript per million mapped reads (RPKM) for higher reliability. Therefore, FPKM was calculated using StringTie for RNA quantification normalization. DESeq2 was used for differential expression analysis, with significantly differentially expressed genes being filtered under the criteria of an adjusted p-value (padj) ≤ 0.05 and log2FoldChange ≥ 2 (Love et al., 2014). For genes with significant differential expression, clusterProfiler was employed for GO and KEGG pathway enrichment analyses (Wu et al., 2021).

### 2.5 Construction of co-expression networks

The FPKM values of significantly differentially expressed genes were extracted to construct a differential expression matrix. The construction and analysis of the co-expression network were conducted using the online tool imageGP (Chen et al., 2022) (https://www.bic.ac.cn/BIC/#/).

### 2.6 Quantitative real-time PCR

Leaf RNA was extracted using the RNAprep Pure Plant Kit (DP432) provided by Tiangen Biotech (Beijing) Co., Ltd (Beijing, China). Real-time fluorescence quantitative analysis was then performed using the real-time quantitative reagent kit (R223) supplied by Vazyme Biotech Co., Ltd (Nanjing, China). First, the RNA was mixed with the reverse transcription system and placed in a PCR instrument (LongGene A300, Hangzhou, China) to obtain cDNA. The synthesized cDNA was then combined with the qRT-PCR reaction system for quantitative expression analysis using a fluorescence quantitation instrument (Analytik Jena qTOWER3G, Jena, Germany). The specific amplification conditions were initial denaturation at 95°C for 3 min, followed by 40 cycles of denaturation at 95°C for 5 s, and annealing at 60°C for 30 s. The relative expression levels were calculated using the Livak method. The experiment was conducted with three biological replicates. *OsRac1* was the internal reference gene (Heid et al., 1996).

## 3 Results

### 3.1 Salt stress effects on pollen in young rice panicles

**Figure 1:**
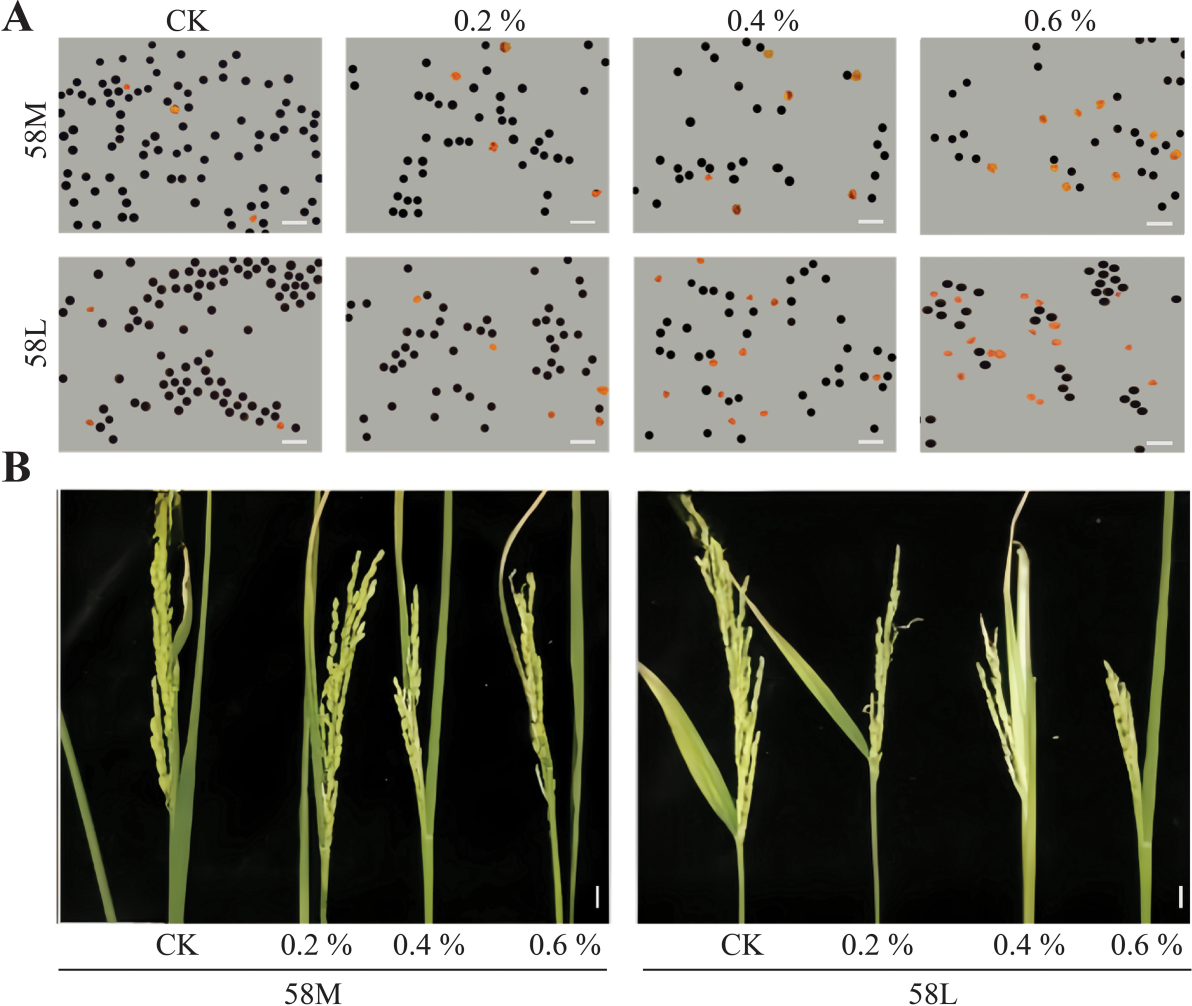
Pollen viability and young panicle morphology of 58M and 58L rice varieties under different NaCl treatments. **(A)** Microscopic images of pollen from 58M and 58L under control (CK), 0.2%, 0.4%, and 0.6% NaCl treatments. The scale bar represents 100 μm. **(B)** Morphological changes in 58M and 58L young panicles under the different treatments (CK, 0.2%, 0.4%, and 0.6% NaCl). The scale bar represents 1 cm. 58M maintained a more robust panicle structure under salt stress than 58L, showing higher tolerance to increasing NaCl concentrations.

It is generally understood that rice is sensitive to salt stress during the reproductive stage, with pollen fertility continuously declining as salt concentration increases (Sarhadi et al., 2012). Under a microscope, normal pollen appears plump and dark brown, whereas sterile pollen is distorted and shrunken, turning yellowish (represented by the yellow particles in the image) (**Figure 1A**). Following statistical counting, it was found that after treatment with 0.6% salt concentration, pollen sterility reached its peak. In 58M and 58L pollen, the sterility rates were 23.07% and 32.67%, respectively, increasing by 15.60% and 25.12% compared to the control. From this, it can be seen that, under salt stress, 58M exhibits a higher tolerance than 58L and has an increased ability to produce viable pollen. At the same time, the morphology of the young panicles was observed (**Figure 1B**) and 58M maintained a shape similar to that of the control group at a salt concentration of 0.2%. It was not until 0.4% and 0.6% salt concentrations that it showed a smaller shape, slower development, and apparent degradation of spikelets compared with the control group. Meanwhile, 58L showed apparent development inhibition at a salt concentration of 0.2%. As the salt concentration increased, the shape became smaller, the development inhibition and spikelet degradation were more prominent, and the degree of influence was significantly higher than that of 58M, which also showed that the salt tolerance of 58M was higher than that of 58L.

### 3.2 RNA sequencing analysis

**Figure 2:**
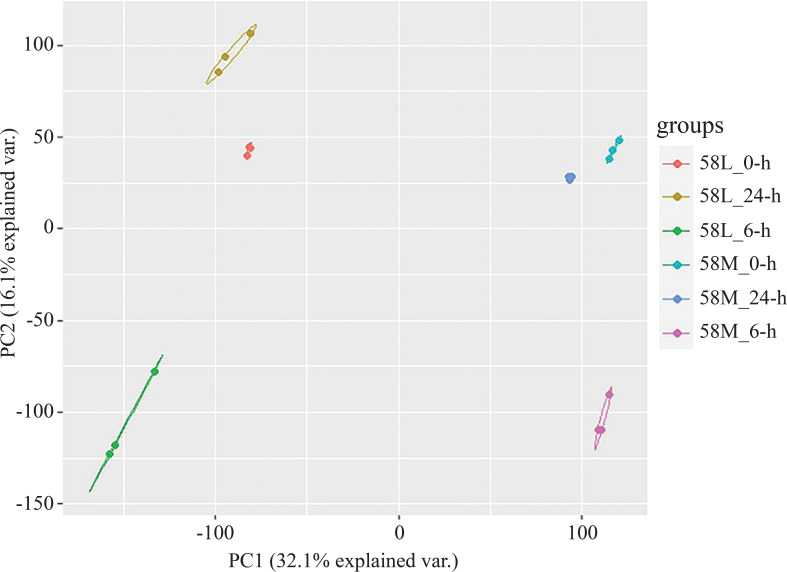
PCA of 18 RNA-Seq samples.

A total of 36 RNA-Seq datasets were obtained from two types of materials under three salt treatment conditions, producing 65.3 GB of raw data. Data filtering using trim_galore resulted in 61.1 GB of clean data. Reference genome alignment was conducted using hisat2, with an average alignment rate above 94%. The FPKM values of all genes from all 18 materials were extracted to form a gene expression data matrix, and principal component analysis (PCA) was conducted using R, with the results visualized. PCA revealed that the three replicates of each material under different treatments clustered together, and the differences between treatments were more significant than those between materials (**Figure 2**). Overall, the high correlation between replicates indicates their high consistency. Several genes were randomly selected for qRT-PCR analysis in three independent replicates to confirm the accuracy of the transcriptomic expression profiles. The results were similar to the expressions observed in the RNA-Seq data, confirming the reliability of the RNA-Seq data for further analysis.

### 3.3 Differential expression analysis

**Figure 3:**
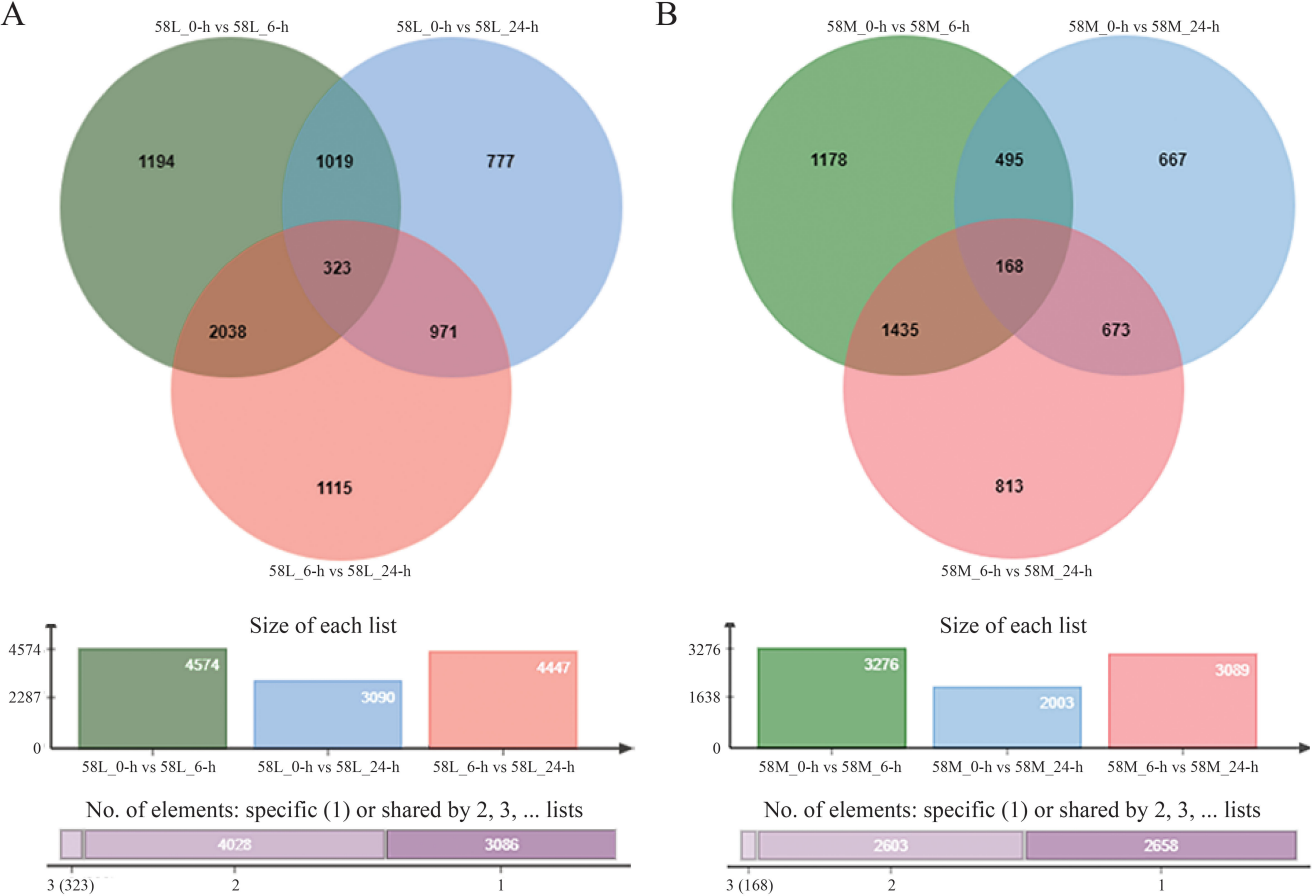
Venn diagrams show the differentially expressed genes (DEGs) in 58L and 58M rice varieties under salt stress. **(A)** DEGs in 58L across different time points (0 h, 6 h, and 24 h) under the 0.6% NaCl treatment when the young panicle length reached 2 cm. **(B)** DEGs in 58M under the same conditions. The overlapping regions indicate the common DEGs between the different time points for each variety.

The expression analysis of significantly different genes was initially conducted for the same material under different salinity conditions (**Figure 3**). There were 2,361 DEGs between 58L_0-h vs. 58L_6-h and 58L_6-h vs. 58L_24-h, 1,342 DEGs between 58L_0-h vs. 58L_6-h and 58L_0-h vs. 58L_24-h, and 1,294 DEGs between 58L_0-h vs. 58L_24-h and 58L_6-h vs. 58L_24-h. There were 1,603 DEGs between 58M_0-h vs. 58M_6-h and M_6-h vs. 58M_24-h, 663 DEGs between 58M_0-h vs. 58M_6-h and 58M_0-h vs. 58M_24-h, and 841 DEGs between 58M_0-h vs. 58M_24-h and 58M_6-h vs. 58M_24-h.

**Figure 4:**
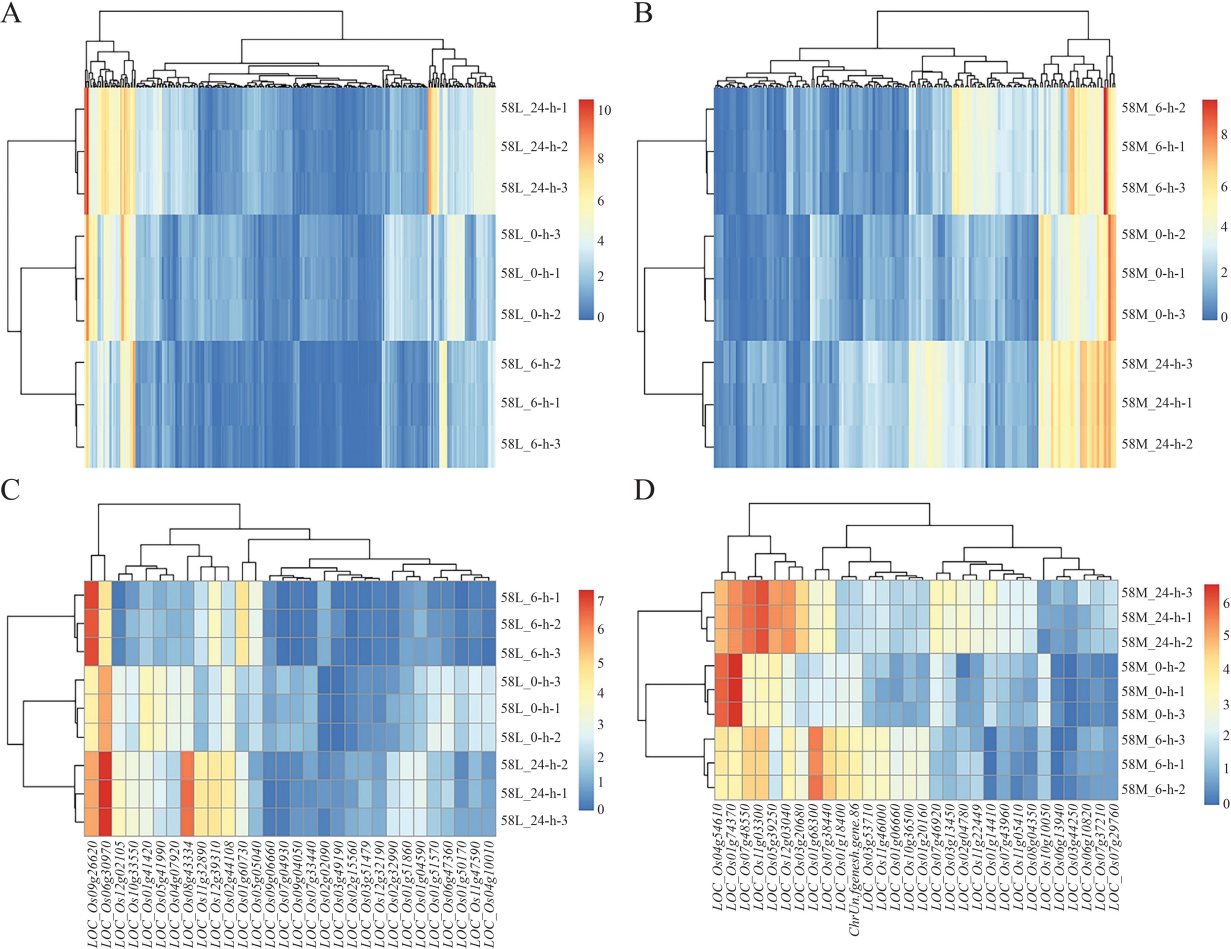
Heatmaps of the differentially expressed genes (DEGs) in 58L and 58M under salt stress at different time points. **(A)** Clustering heatmap of DEGs in 58L across 0 h, 6 h, and 24 h under the 0.6% NaCl treatment when the young panicle length reached 2 cm. **(B)** Clustering heatmap of the DEGs in 58M under the same conditions. **(C)** Clustering heatmap of the top 30 DEGs in 58L across the different time points. **(D)** Clustering heatmap of the top 30 DEGs in 58M across the different time points. Red indicates upregulated genes, while blue indicates downregulated genes. These heatmaps illustrate the dynamic transcriptional responses of 58L and 58M to salt stress over time.

Using pheatmap, a clustering analysis was performed separately on 323 common significant DEGs in 58L and 168 in 58M. The results showed a distinct area of high expression in red on the left side for l-24, indicating that some genes in 58L were significantly upregulated after 24 hours of treatment. Simultaneously, some parts in the middle turned bluer than 58L_0-h, indicating a significant downregulation of some genes. Similar changes occurred in 58L_6-h but were less pronounced (**Figure 4A**). For 58M_24-h, there was a relatively uniform high expression area on the right side, and 58M_6-h also had a slightly narrower high expression area on the right, indicating that these genes in 58M were highly expressed under salt stress (**Figure 4B**).

Analysis of the top 30 DEGs revealed that in 58L, *LOC_Os06g30970* and *LOC_Os09g26620* were significantly highly expressed in both 58L_6-h and 58L_24-h, indicating their vital role in the salt stress response of 58L. Additionally, *LOC_Os02g44108*, *LOC_Os12g39310*, *LOC_Os11g32890*, and *LOC_Os08a43334* showed higher expression in 58L_24-h, suggesting they may play a more significant role under prolonged high salt stress conditions in 58L (**Figure 4C**). In 58M, *LOC_Os01g74370* and *LOC_Os04g54610* exhibited high expression under no-stress conditions, with a significant decrease in short-term stress and a slight decrease in long-term stress. This pattern indicates that these genes in 58M initially undergo a significant downregulation, followed by a slight upregulation of expression as the duration of salt stress increases. Furthermore, *LOC_Os11g03300* and *LOC_Os07g48550* showed progressively increasing expression with prolonged salt stress in 58M, indicating their significant role under long-term stress (**Figure 4D**). At the same time, some genes exhibited a pattern of upregulation under short-term stress and downregulation under long-term stress, suggesting that the expression activity of some genes in 58M was difficult to maintain over a prolonged period under high salt conditions.

**Figure 5:**
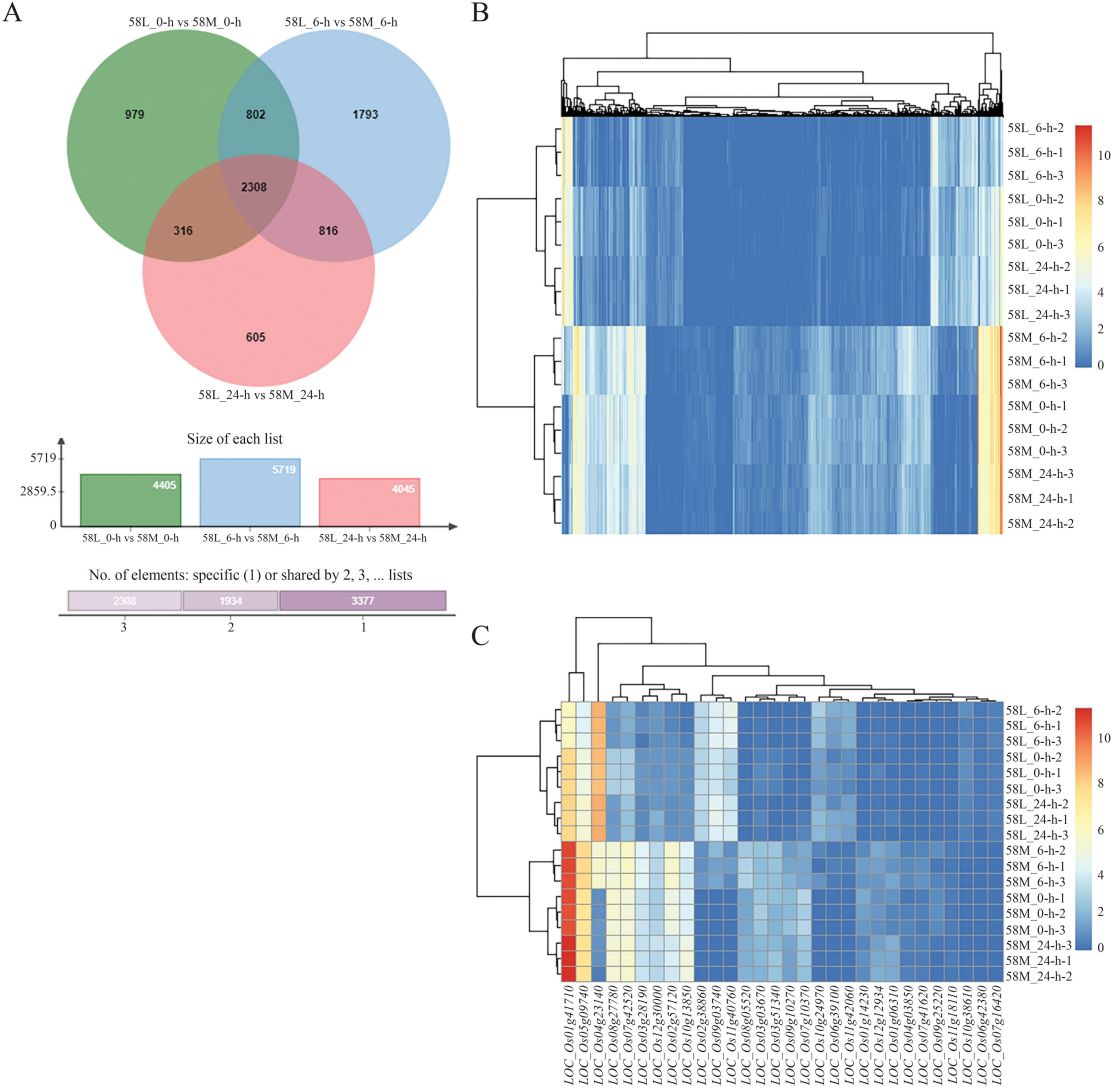
Venn diagram and heatmaps showing differentially expressed genes (DEGs) between the 58M and 58L rice varieties under salt stress at different time points. **(A)** Venn diagram of the DEGs between 58M and 58L across 0 h, 6 h, and 24 h under the 0.6% NaCl treatment when the young panicle length reached 2 cm. **(B)** Clustering heatmap of all the DEGs across both varieties and all treatments. **(C)** Clustering heatmap of the top 30 DEGs across both varieties and all treatments. Red indicates upregulated genes, while blue indicates downregulated genes, highlighting the distinct transcriptional profiles of 58M and 58L under salt stress.

Differential expression analysis between different varieties under the same treatment showed 3,110 DEGs between 58L_0-h vs. 58M_0-h and 58L_6-h vs. 58M_6-h, 2,624 DEGs between 58L_0-h vs. 58M_0-h and 58L_24-h vs. 58M_24-h, and 3,124 DEGs between 58L_6-h vs. 58M_6-h and 58L_24-h vs. 58M_24-h. There were 2,308 common DEGs across the three treatments, far more than the gene expression differences between different treatments of the same variety, indicating significant differences in salt tolerance between 58L and 58M (**Figure 5A**).

Using pheatmap, clustering analysis of the 2,308 differentially expressed DEGs showed a very clear high expression area on the right side for 58L, further proving significant differences in salt tolerance between 58L and 58M (**Figure 5B**). Analysis of the top 30 DEGs showed that 58M had significantly high expression in *LOC_Os05g09740* and *LOC_Os01g41710*, while *LOC_Os04g23140* was highly expressed in 58L (**Figure 5C**). Additionally, several blocks in the middle indicate differences in gene expression regulation between 58L and 58M in response to salt stress.

### 3.4 Enrichment analysis of differentially expressed genes

**Figure 6:**
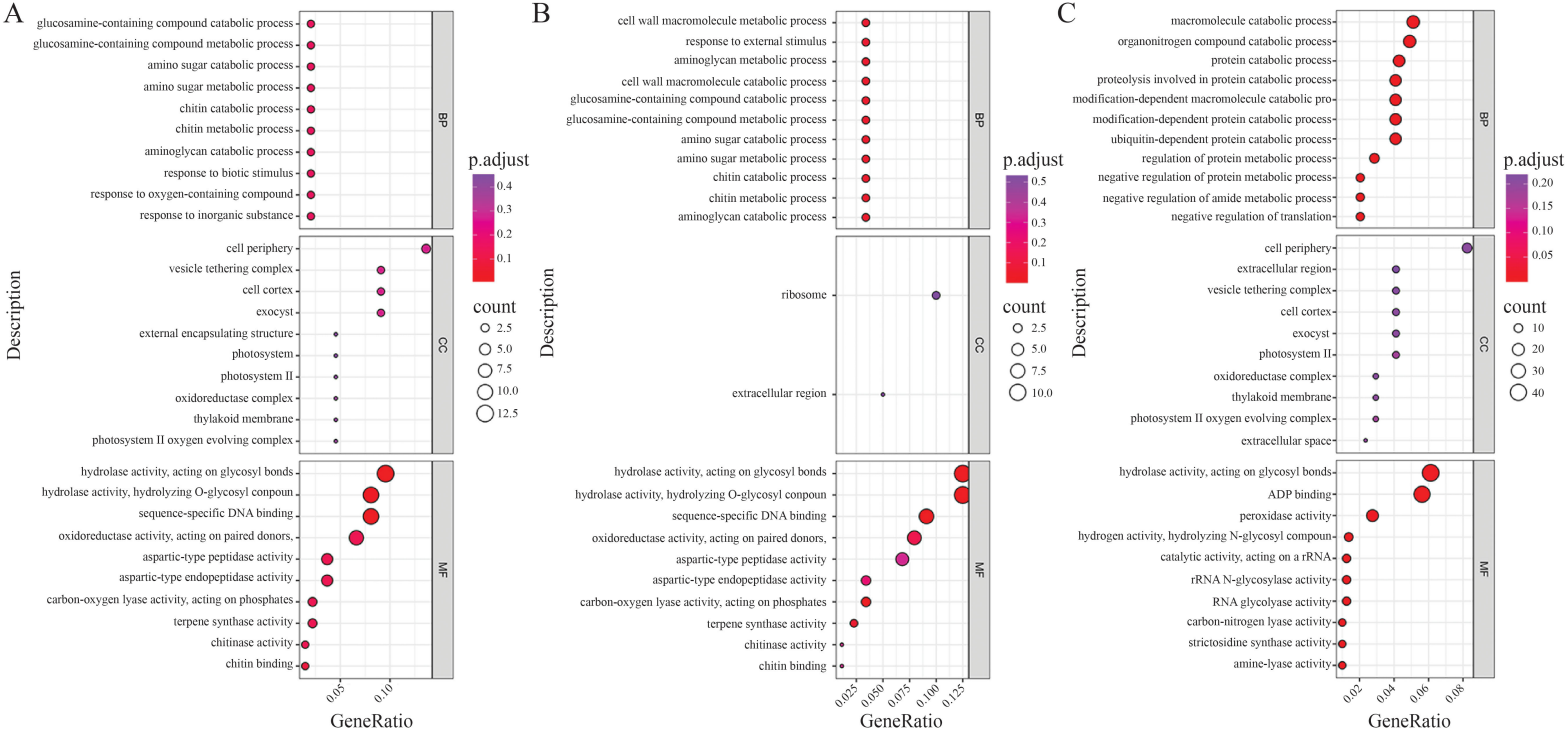
Gene Ontology (GO) enrichment analysis of the differentially expressed genes (DEGs) under salt stress. The size of the bubbles represents the number of genes involved, and the color gradient from purple to red indicates the significance level of enrichment (adjusted p-value). **(A)** GO enrichment analysis of the DEGs in 58L across different time points (0 h, 6 h, and 24 h) under the 0.6% NaCl treatment. **(B)** GO enrichment analysis of the DEGs in 58M under the same conditions. **(C)** GO enrichment analysis of the DEGs across both varieties and all treatments, highlighting common biological processes affected by salt stress.

Enrichment analysis was conducted using R, referencing publicly available rice species annotation packages. Gene Ontology analysis of the DEGs from the same material under different treatments indicated that specific DEGs in 58L were significantly enriched in GO terms, including the degradation and metabolism of glucosamine-related compounds, degradation and metabolism of aminosugars, degradation and metabolism of chitin, degradation of aminosugar uronic acids, response to biological stimuli, response to oxygen-containing compounds, and response to inorganic substances (**Figure 6A**). Additionally, significant GO terms enriched in 58M DEGs included the degradation and metabolism of cell wall macromolecules, response to external biological stimuli, degradation and metabolism of aminosugar uronic acids, degradation and metabolism of glucosamine-related compounds, degradation and metabolism of aminosugars, and degradation and metabolism of chitin (**Figure 6B**).

GO analysis of the DEGs between different materials under each treatment showed significant enrichment in GO terms related to the degradation of organic nitrogen compounds, protein degradation processes and the associated hydrolytic reactions, degradation processes of modified proteins, ubiquitin-dependent protein degradation processes, negative regulation of protein metabolic processes, negative regulation of amide metabolic processes, and negative regulation of translation processes (**Figure 6C**).

**Figure 7:**
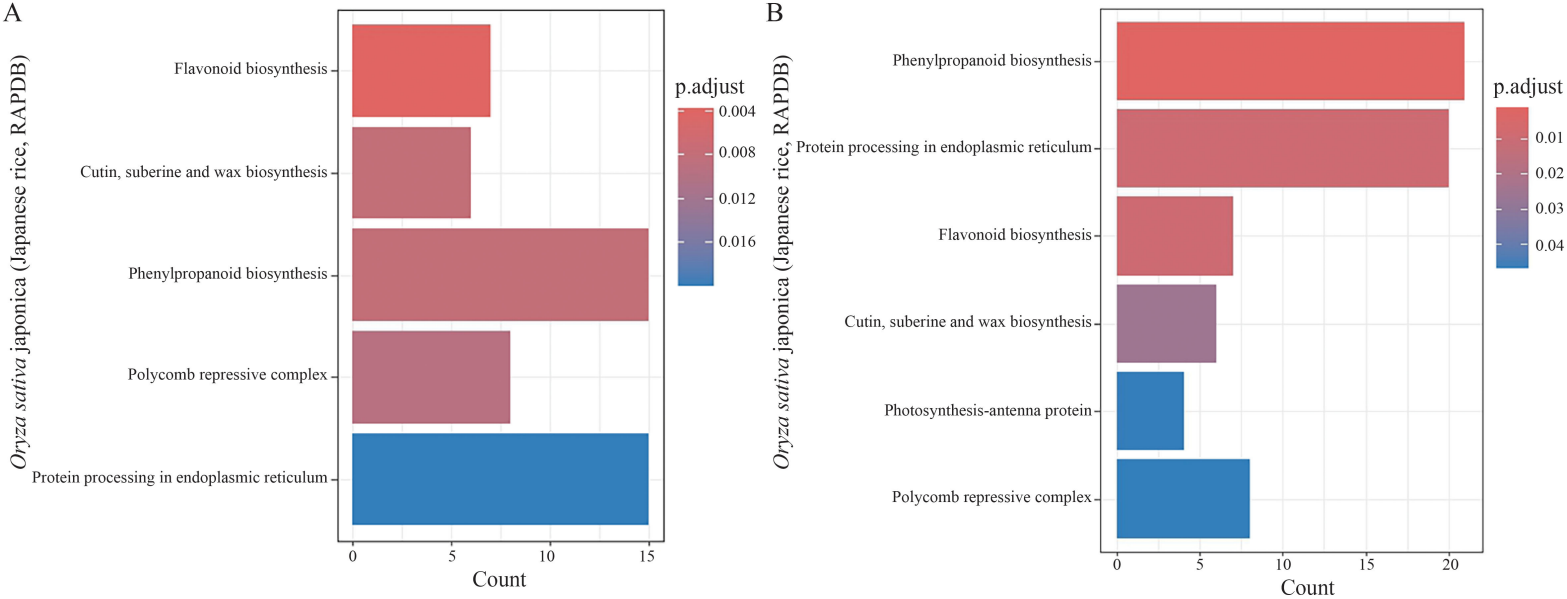
Kyoto Encyclopedia of Genes and Genomes (KEGG) pathway enrichment analysis of the differentially expressed genes (DEGs) under salt stress. The horizontal bar charts represent the enriched pathways, with the bar color transitioning from red to blue indicating the significance level (adjusted p-value). **(A)** KEGG enrichment analysis of the DEGs in both 58M and 58L across different time points (0 h, 6 h, and 24 h) under the 0.6% NaCl treatment. **(B)** KEGG enrichment analysis of the DEGs across both varieties and all treatments shows the key metabolic and signaling pathways involved in the response to salt stress.

KEGG pathway enrichment analysis of the DEGs between different materials under each treatment revealed pathways including biosynthesis of flavonoids, biosynthesis of cutin, suberin, and wax, biosynthesis of phenylpropanoids, biosynthesis of polycomb inhibitory complex, and protein processing in the endoplasmic reticulum (**Figure 7A**). Additionally, KEGG pathway enrichment analysis of the DEGs between different treatments for each material highlighted biosynthesis of phenylpropanoids, protein processing in the endoplasmic reticulum, biosynthesis of flavonoids, biosynthesis of cutin, suberin, and wax, photosynthesis processes related to antenna proteins, and biosynthesis of polycomb inhibitory complex (**Figure 7B**).

### 3.5 Exploration in the weighted gene co-expression network

**Figure 8:**
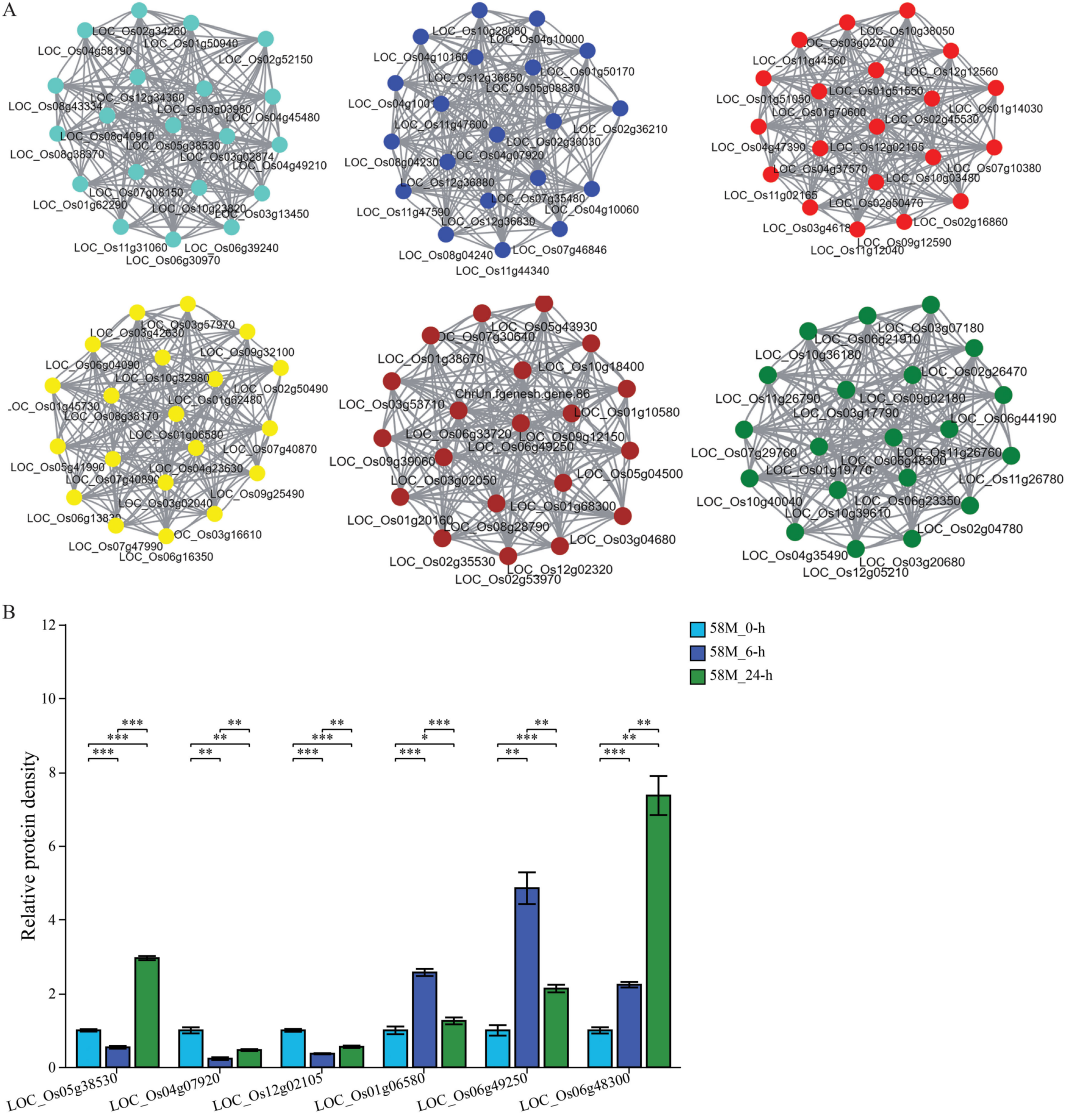
Co-expression network analysis and relative expression levels of the key salt tolerance genes. **(A)** The co-expression network shows six distinct modules, each containing a core gene. These core genes potentially affect salt tolerance in the 58M and 58L rice varieties. **(B)** Bar plots showing the relative expression levels of core genes across different time points (0 h, 6 h, and 24 h) under the 0.6% NaCl treatment compared to the control at 0 (h) The significance of differences in expression between treatments was assessed using t-tests, with significance levels indicated as follows: *p < 0.05, **p < 0.01, ***p < 0.001.

WGCNA was conducted on a total of 469 significantly differentially expressed genes between treatments for each material, constructing a co-expression network related to rice salt tolerance (with a soft-thresholding power of 18 and R-squared > 0.85). This analysis involved six expression modules, each identifying core, peripheral, and edge genes (**Figure 8A**). Genes such as *LOC_Os05g38530*, *LOC_Os04g07920*, *LOC_Os12g02105*, *LOC_Os01g06580*, *LOC_Os06g49250*, and *LOC_Os06g48300* are potential candidates related to rice salt tolerance. The gene expression levels of six core genes were extracted from the transcriptome sequencing data. The analysis revealed that most of these genes underwent significant expression changes under different treatments, indicating that these genes are closely related to rice salt tolerance and are likely potential genes for rice salt tolerance (**Figure 8B**).

### 3.6 Quantitative real-time PCR

**Figure 9:**
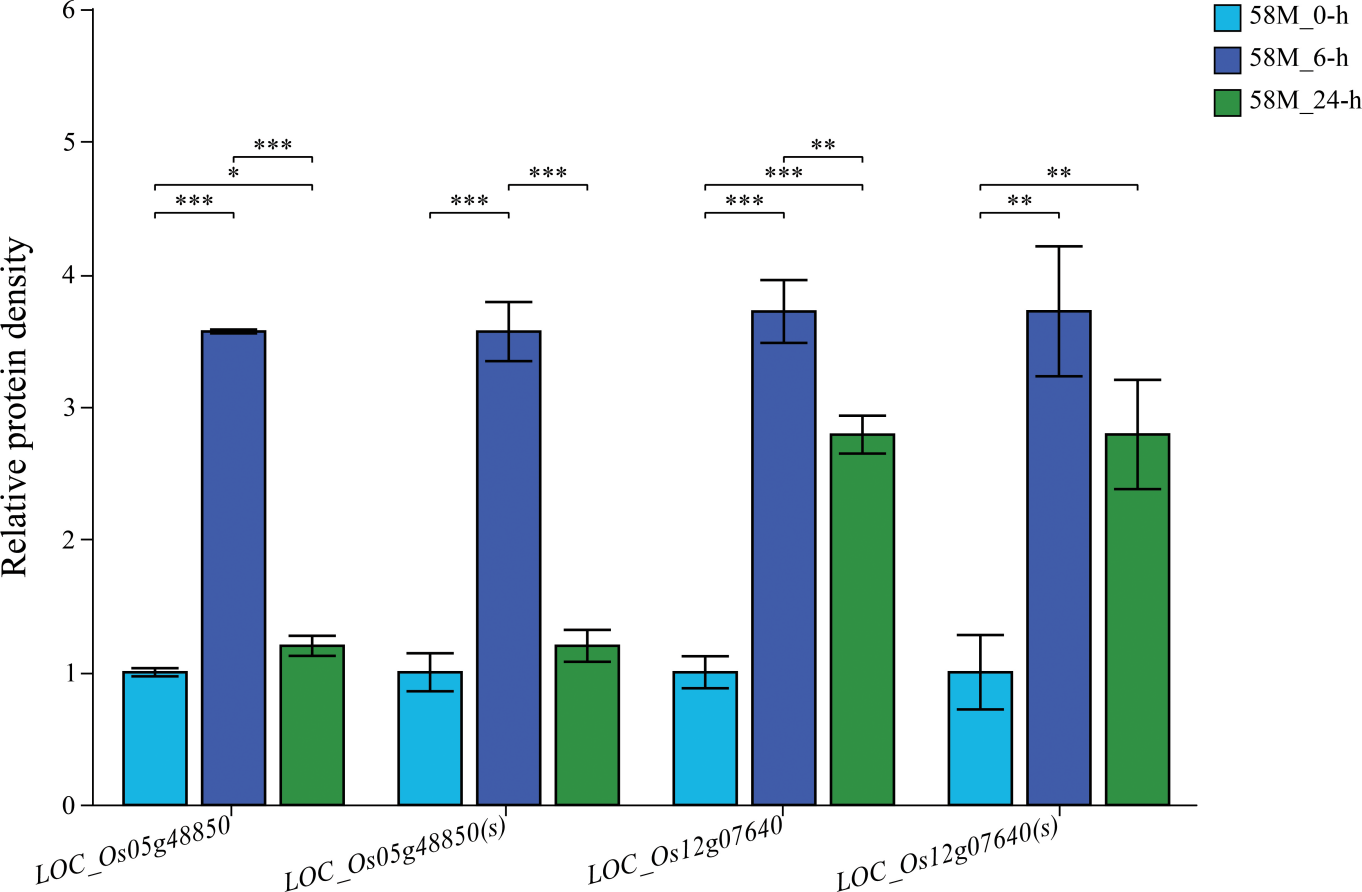
Validation of differentially expressed genes (DEGs) through qRT-PCR and RNA-seq analysis. Bar plots show the relative expression levels of two randomly selected DEGs across different time points (0 h, 6 h, and 24 h) under the 0.6% NaCl treatment, as measured by qRT-PCR [indicated by the gene names followed by “(s)”)]. The expression levels from the RNA-seq data for the same genes are also plotted for comparison. Each experiment was performed in three biological replicates, and the relative expression was calculated for the control at 0 h. The significance of expression differences between treatments was assessed using t-tests, with significance levels as follows: *p < 0.05, **p < 0.01, ***p < 0.001.

Two significantly differentially expressed genes were randomly selected for qRT-PCR analysis. The results showed that the relative expression level of *LOC_Os05g48850* increased by 4.1-fold after 6 hours of salt treatment and then decreased to 1.3-fold after 24 h, demonstrating high sensitivity during the salt treatment process and low sustainability of high expression levels after prolonged salt exposure. Additionally, the expression of *LOC_Os12g07640* sharply increased to 3.8-fold after 6 h of salt treatment. Then, it decreased to 2.5-fold after 24 h, indicating high sensitivity to salt treatment and higher sustainability of expression levels after prolonged exposure (**Figure 9**). The expression patterns of *LOC_Os05g48850* and *LOC_Os12g07640* were broadly consistent with the transcriptome analysis data, confirming the reliability of the transcriptomic data. Furthermore, the sensitivity of *LOC_Os05g48850* and *LOC_Os12g07640* during the salt stress process suggests that they may play regulatory roles in rice salt stress. These studies provide a theoretical basis for a deeper understanding of the molecular mechanisms of salt stress in rice and offer new genetic resources for research on rice salt tolerance.

## 4 Discussion

### 4.1 Impact of salt stress on rice morphology and fertility

Research indicates that salt stress can cause the closure of stomata in rice leaves, altering leaf water potential, transpiration rate, leaf temperature, and relative leaf water content, leading to a reduction in the photosynthetic area available to support sustainable growth (Mohanty et al., 2023). Different rice varieties exhibit varying responses to salt stress. Some can maintain better traits under high salt conditions, preserving specific morphologies and cellular structures and demonstrating higher salt tolerance. In contrast, others are more susceptible to ion toxicity, osmotic stress, and ROS damage under high salt conditions. These differences may be attributable to genetic variations among varieties and the plant’s adaptability to high salt stress (Arajmand et al., 2020). Studies have shown that in some rice varieties, sterility of the spikelet due to salinity effects and nutrient deficiencies can lead to reduced seed setting rate, pollen carrying capacity, and pollen activity (Abdullah et al., 2001). This may be due to a lack of carbohydrates, reduced transfer of soluble sugars, and inhibited starch synthase activity. Additionally, salt stress leads to an accumulation of abscisic acid (ABA) in the plant, thereby regulating the clearance of ROS, ion homeostasis, and stomata closure under salt stress (Ithal and Reddy, 2004). Moreover, the intense competition between Na^+^ and Cl^-^ against K^+^, Ca^2+^, and NO^3-^ can cause an ion imbalance, triggering the salt overly sensitive pathway (SOS) for salt stress signaling to enhance Na^+^ tolerance. Under different salt treatment conditions, we observed changes in pollen appearance and fertility changes in the young rice panicles. We found apparent morphological and genetic differences among different rice varieties under high salt conditions. The results of our study demonstrate that salt stress significantly affects rice morphology and fertility, with notable differences between the two rice varieties, 58M and 58L. Under increasing salt concentrations, 58M exhibited a higher pollen viability and better panicle development than 58L. This aligns with previous studies that show salt stress can severely reduce pollen fertility, leading to sterility and decreased yield potential. The morphological differences observed in the young panicles are consistent with the ion toxicity and osmotic stress effects caused by salt stress. These findings suggest that 58M possesses inherent genetic traits that confer greater resilience to salt-induced damage, especially during critical reproductive stages.

### 4.2 Differentially expressed genes in response to salt stress

In studying plant abiotic stress, salt stress has always been a widely focused on topic in plant biology and agricultural science. With the continuous increase in the global population and the growing severity of food security issues, how to cultivate food crops in saline-alkali soils has gained considerable attention and numerous research advancements have been achieved, which are crucial for understanding the intrinsic mechanisms of plant salt stress. The salt overly-sensitive pathway is one of the more thoroughly researched salt stress signal transduction pathways (Gupta et al., 2021). The calcium-binding protein *OsSOS3* perceives changes in the cytosolic calcium signal caused by salt stress and then interacts with *SnRK3*. *SnRK3* activates *OsSOS2* and *OsCIPK24*, which phosphorylate *OsSOS1* (a Na^+^/H^+^ antiporter on the plasma membrane), thereby controlling Na^+^ homeostasis and enhancing rice tolerance to salt stress. Additionally, some studies have shown that plant hormones undergo significant changes in plant salt signal transduction, including ABA, ethylene (ETH), jasmonic acid (JA), gibberellins (GA), cytokinins (CK), and salicylic acid (SA). These hormones work in an integrated and coordinated manner to regulate plant growth and mediate plant responses to abiotic stress (Khan et al., 2023). For instance, *OsNAC2* decreases the biosynthesis and content of auxin while increasing CK biosynthesis and content, thereby integrating the auxin and CK pathways to regulate the development of rice roots under normal and saline water conditions (Zhao et al., 2023). Through enrichment analysis, we have identified the significant role of ubiquitin-mediated protein degradation, the negative regulation of protein and amide metabolic processes, the biosynthesis of flavonoids, and the biosynthesis of cutin, suberin, and wax in the salt tolerance process of rice. RNA-seq analysis revealed significant gene expression differences between the two rice varieties under different salt stress conditions. A total of 2,308 DEGs were identified between 58M and 58L, with many genes related to phenylpropanoid biosynthesis, flavonoid biosynthesis, and protein processing in the endoplasmic reticulum. These pathways are vital for enhancing plant stress tolerance by stabilizing cell walls and mitigating oxidative stress. Identifying these pathways supports previous research linking these biological processes to improved plant salt tolerance. Furthermore, after more extended periods of salt treatment in different varieties, some genes exhibited fluctuating expression, indicating variability in signal transduction and adaptive stress responses in rice when encountering salt stress. Overall, the phenotypic characteristics of young rice panicles under salt stress and the dynamic changes in differential gene expression reflect a plant’s response and resistance level to salt stress.

### 4.3 Co-expression network analysis and identification of key salt tolerance genes

Co-expression networks utilize the characteristics of functionally related genes in biological cells that are co-expressed in a coordinated manner under specific conditions. These relationships are represented as a network by computing the correlation coefficient matrix between the genes and setting a threshold to filter correlations. This approach offers a perspective for studying gene interactions and regulatory relationships among thousands of genes in expression data generated by high-throughput sequencing technologies (Chen et al., 2024). Using WGCNA, four highly significant gene modules were identified in grapes from the transcriptome data of grape leaf samples under different temperatures. A total of 20 heat stress-responsive genes were discovered, including four genes with higher expression levels than those in heat-sensitive materials, making them potential positive regulators of heat tolerance in grapes (Wu et al., 2023). In our study, using WGCNA on the DEGs between varieties and treatments, six highly significant gene expression modules were identified, uncovering 42 salt stress-responsive genes. qRT-PCR analysis showed that the expression of these genes initially increased and then decreased with the duration of salt treatment, indicating their phased regulation. Functional annotation of six core genes in the co-expression network revealed that *LOC_Os05g38530* encodes a heat shock protein involved in responses to abiotic stimuli and stress in various organelles, *LOC_Os04g07920* encodes an expressed protein whose function is still under investigation, and *LOC_Os12g02105* encodes a lipid transfer protein (LTP) precursor involved in transport functions on the cell membrane. Furthermore, *LOC_Os01g06580* encodes a protein with a bundling protein domain function in the cell membrane and plasmalemma, *LOC_Os06g49250* encodes a peptide transporter (PTR2) that executes transport functions in the cell membrane and vacuoles, and *LOC_Os06g48300* encodes a protein phosphatase (2C) involved in protein modification, metabolic processes, and stress responses on the plasma membrane. Although the roles of these genes in rice salt tolerance still require further validation, subsequent studies using biotechnological methods can delve deeper into elucidating their molecular mechanisms.

## 5 Conclusions

This study revealed the developmental characteristics of rice panicles and the differential expression patterns of related genes under salt stress by analyzing the phenotypes and transcriptomes of two rice varieties, 58M and 58L, under different salt stress conditions. Unlike previous studies, this study combined phenotype observation with RNA Seq high-throughput sequencing technology. It constructed a gene co-expression network related to salt stress using weighted gene co-expression network analysis. This multi-level approach had unique advantages in revealing the molecular mechanisms of rice under salt stress. We identified six specific gene modules highly associated with rice salt stress through the construction of a co-expression network and screened out six potential candidate genes, namely, *LOC_Os05g38530, LOC_Os04g07920, LOC_Os12g02105*, *LOC_Os01g06580, LOC_Os06g49250*, and *LOC_Os06g48300*. These genes may play a key role in plant response to salt stress. These genes provide new genetic resources for studying salt tolerance in rice and lay the foundation for further exploring its molecular mechanisms. These findings offer essential candidate gene targets for improving rice varieties and enhancing salt stress tolerance. In addition, this study used qRT PCR to validate the reliability of the RNA-Seq data, and the results showed that the expression trend of the genes was consistent with transcriptome analysis, further demonstrating the accuracy of our data. This method, which combines transcriptomic analysis, co-expression network construction, and experimental verification, provides a new approach for comprehensively analyzing the salt stress response of rice and provides a theoretical basis and practical reference for future molecular breeding research on rice stress resistance. Through this study, we revealed the molecular regulatory network of rice under salt stress conditions. We provided new potential functional genes and their roles in salt stress response, which are discoveries and necessary supplements to existing research on rice salt resistance.

## Data Availability

The original contributions presented in the study are publicly available. This data can be found here: National Genomics Data Center (NGDC) Genome Sequence Archive (GSA), accession CRA017107.
